# Annotations

**Published:** 1898-09-10

**Authors:** 


### Annotations.

### Provident Dispensaries.

We have often had occasion to criticise the proceedings and the aspirations of the Medical Reform Association. It is then with considerable pleasure that we call attention to the very useful discussion on provident dispensaries which took place at the meeting of the Association recently held in Edinburgh. At that meeting two papers were read, one by Dr. Otto Wunderlich and the other by Dr. Richardson Rice, both of which displayed considerable practical grasp of the subject. The most notable and important suggestion in the paper by Dr. Wunderlich is that, in arranging the fees, instead of insisting upon a wage limit, and thus drawing a strict line which must appear harsh to those adversely affected by it, the fees should be made to vary with the income or wages of the members, "in the same manner as the medical tariff varies with the value of a patient's house." This gets rid of the fixed and rigid line and of the endless exceptional cases which claim exemption from it. Dr. Wunderlich says, "The rate of 4s. a year is fair only for the pauper class and persons earning less than 20s. or 25s. a week. A rate of 6s. to 8s. a year might be held sufficient for persons earning up to 30s. and 40s. a week, whilst a rate of 10s. a year would suffice for any person whatsoever who did not mind putting up with the restrictions and inconveniences attendant upon provident work." Without pinning ourselves down to the particular figures, one can hardly avoid seeing that if the medical men in a town were decently unanimous, a scheme of this sort might be worked without hardship to anyone. Dr. Richardson Rice's paper was important, inasmuch as it described the actual working of a provident scheme, managed by the medical men themselves, to provide medical relief for those whose weekly earnings are under 30s. He very truly says that the exigencies of professional life render it inevitable that some movement of this nature should be started, but he properly maintains that in any organisation of the sort no middleman must come between medical men and their patients. "If the medical men in every town will only band themselves together in some agency for the supply of medical relief to the working classes, they will be taking the most important step in breaking the power of" irregular institutions, in which medical men are exploited by lay committees. This is constructive reform, something far better than mere protests against medical aid associations.

### Water Supply and Public Health.

The report of the Local Government Board inspectors in regard to the outbreak of typhoid fever at Maidstone last year contains certain remarks as to the duties of sanitary authorities in relation to water supply which serve well to show the muddle into which this most important detail of sanitary administration has been allowed to fall. It is very properly pointed out that the history of the epidemic raises questions as to how far the regulations of the Board relating to the duties of medical officers, and the statutes which regulate the powers and obligations of private water companies, are sufficient to ensure a reasonable amount of protection to the public health. Truly, the general order of the Board of 1891 prescribes that the medical officer of health "shall inform himself, as far as may be practicable, respecting all influences affecting, or threatening to affect injuriously, the public health within the district," and this might be held to make it his duty to visit and inspect the works of a private company supplying water to his district even though the works were situated outside it, as is the case at Maidstone. Moreover, section 7 of the Public Health (Water) Act, 1878, imposes upon rural sanitary authorities the duty of taking such steps as may be necessary to ascertain the condition of the water supply within their districts, yet, notwithstanding all this, they have no legal right of access to the works of the company. In this respect indeed London is no better off than the country. It seems to us that Parliament is directly responsible for the contradiction between these various Acts, and that Parliament may fairly be asked to give to the sanitary authorities the power which is required for the performance by them of the duties imposed upon them.

### War in Hot Countries.

The reports which continue to arrive regarding the distressing effects of the exposure and hard work to which the American troops were exposed in Cuba, serve to emphasise the importance of the principles which, as the result of large experience, English and Anglo-Indian authorities have been led to adopt in the conduct of war in the tropics. No one can fail to admire the alertness with which the United States set itself to the herculean task of suddenly developing a great army of upwards of 150,000 men, nor is it easy for those who know how complex a machine an army really is, to refrain from expressing surprise at the measure of success which even so great a nation was able to attain in so quickly getting together so large a fighting force. But the feat was too great. Although the object was gained, the loss was considerable, and it is beginning to be seen that the English plan of conducting warfare in the tropics, a plan which has again and again drawn upon us the ridicule of continental writers is after all the best. Some military critics are never tired of poking fun at our enormous baggage trains and the number of our camp followers, but with the end of the war in Cuba it is being asked in many quarters whether the sufferings of the American troops before Santiago were really inevitable. English experience seems to suggest that they were not, and amid the general cry of indignation which is arising in the States in regard to many of the details of the campaign, especially those concerned with transport and commissariat, we note a remark in the *Philadelphia Press* which points to English experience, and says, "White men, the English have learned, cannot campaign in the open air during the wet season in the tropics unless they have an army of attendants," and goes on to add "The fighting man in the tropics must be taken care of." That is, indeed, just the secret of success. It is, no doubt, enormously costly, but disaster is more costly still, and the chance of disaster hangs continually over every army which takes the field in tropical countries unless the medical department has free play, and the transport is arranged on an efficient scale, whatever the expense and the difficulty may be.

